# Considerations in the use of different spirometers in epidemiological studies

**DOI:** 10.1186/s12940-019-0478-2

**Published:** 2019-04-25

**Authors:** Edith B. Milanzi, Gerard H. Koppelman, Marieke Oldenwening, Sonja Augustijn, Bernadette Aalders-de Ruijter, Martijn Farenhorst, Judith M. Vonk, Marjan Tewis, Bert Brunekreef, Ulrike Gehring

**Affiliations:** 1 Institute for Risk Assessment Sciences (IRAS), Utrecht University, P.O. Box 80178, 3508 TD Utrecht, The Netherlands; 2 University Medical Center Groningen, Department of Pediatric Pulmonology and Pediatric Allergology, Beatrix Children’s Hospital, University of Groningen, Groningen, The Netherlands; 3 University of Groningen, University Medical Center Groningen, Groningen Research Institute for Asthma and COPD (GRIAC), Groningen, The Netherlands; 4 University of Groningen, University Medical Center Groningen, Department of Pulmonary Diseases, Groningen, The Netherlands; 5 Netherlands Expertise Centre for Occupational Respiratory Disorders, Utrecht, The Netherlands; 6 University of Groningen, University Medical Center Groningen, Department of Epidemiology, Groningen, The Netherlands; 7 Julius Center for Health Sciences and Primary Care, University Medical Center Utrecht, Utrecht, The Netherlands

**Keywords:** Calibration, Epidemiological studies, Lung function, Spirometry, Systematic difference

## Abstract

**Background:**

Spirometric lung function measurements have been proven to be excellent objective markers of respiratory morbidity. The use of different types of spirometers in epidemiological and clinical studies may present systematically different results affecting interpretation and implication of results. We aimed to explore considerations in the use of different spirometers in epidemiological studies by comparing forced expiratory volume in 1 s (FEV_1_) and forced vital capacity (FVC) measurements between the Masterscreen pneumotachograph and EasyOne spirometers. We also provide a correction equation for correcting systematic differences using regression calibration.

**Methods:**

Forty-nine volunteers had lung function measured on two different spirometers in random order with at least three attempts on each spirometer. Data were analysed using correlation plots, Bland and Altman plots and formal paired t-tests. We used regression calibration to provide a correction equation.

**Results:**

The mean (SD) FEV_1_ and FVC was 3.78 (0.63) L and 4.78 (0.63) L for the Masterscreen pneumotachograph and 3.54 (0.60) L and 4.41 (0.83) L for the EasyOne spirometer. The mean FEV_1_ difference of 0.24 L and mean FVC difference of 0.37 L between the spirometers (corresponding to 6.3 and 8.4% difference, respectively) were statistically significant and consistent between younger (< 30 years) and older volunteers (> 30 years) and between males and females. Regression calibration indicated that an increase of 1 L in the EasyOne measurements corresponded to an average increase of 1.032 L in FEV_1_ and 1.005 L in FVC in the Masterscreen measurements.

**Conclusion:**

Use of different types of spirometers may result in significant systematic differences in lung function values. Epidemiological researchers need to be aware of these potential systematic differences and correct for them in analyses using methods such as regression calibration.

## Background

Spirometry is a commonly used test of lung function, an important tool in the diagnosis, and monitoring of respiratory diseases and is frequently used in epidemiological and clinical research [1]. Results of spirometry tests depend on several factors including technical factors such as the type of spirometer used, personal factors such as a subject’s posture, and the cooperation between the subject and the technician, which need to be considered in clinical and epidemiological studies.

Despite potential differences between spirometers, there may be compelling reasons to use different spirometers in clinical and epidemiological research. In large-scale multicentre studies for example, for efficiency reasons more than one spirometer of the same type or different spirometers of different types may be used in different centres. In follow-up studies, there may be need to replace older spirometers by newer spirometers.

Comparisons between different types of spirometers as well as similar types of spirometers have been performed in several studies [2–5]. Systematic differences between different types of spirometers have been reported [2, 4]. Such differences can bias exposure-health relationships in studies where the use of a specific spirometer is associated with exposure, e.g. in multi-centre studies of effects of ambient air pollution where different spirometers are used in different study regions with different levels of exposure. Adjustment for type of spirometer is one possibility to account for systematic differences between spirometers. However, this may result in over-adjustment if region is also an important determinant of exposure. Methods such as regression calibration are more suitable in such situations, but require data on comparability of devices [6].

In this study we compared FEV_1_ and FVC measurements from two widely used spirometers - the Masterscreen pneumotachograph and the EasyOne spirometer that were simultaneously used in the Prevention and Incidence of Asthma and Mite Allergy (PIAMA) birth cohort study. We also investigated comparability between two EasyOne spirometers. We used the obtained measurements to provide a correction equation to adjust for differences between the spirometers in an epidemiological study.

## Methods

### Comparison study design and study population

Two series of spirometry tests were performed in volunteers by trained research staff between April and May 2017. In the first test series that we consider to be our main comparison performed at the University Medical Centre Groningen, we compared the Masterscreen pneumotachograph with an EasyOne spirometer (referred to here as EasyOne1). Two highly experienced and trained technicians conducted spirometry measurements in the first test series (one with the Masterscreen pneumotachograph and one with the EasyOne1). We let each technician use a different spirometer by design to reflect a real-life multicentre research setting where different spirometers are used in different centers by different technicians*.* In the second series, one of the technicians involved in the first test series performed the tests at Utrecht University, and the EasyOne1 from the first series was compared to a second EasyOne spirometer of the same generation, referred to as EasyOne2 (both purchased in 2008). In both series, all volunteers performed tests on both spirometers in random order but in immediate succession to eliminate confounding by individual characteristics. Forced expiratory volume in 1 s (FEV_1_) and forced vital capacity (FVC) were measured in sitting position, while wearing a nose clip. Measurements that fulfilled the ATS/ERS criteria [1] were included in the analysis (*n* = 45 for each of the series). In addition, test results were included which did not meet these criteria (difference between the largest and next largest value ≤150 mL for FEV_1_ and FVC), but which were obtained from otherwise technically acceptable flow-volume curves with the difference between the largest and next largest values for FEV_1_ and FVC ≤ 200 mL, (n = 4 for each of the two series) as in previous analyses [7]. Zero flow was established before each measurement with both devices. For each test series, the final study population consisted of 49 volunteers. Information on ethnicity, self-reported weight, height and age of volunteers was also collected.

### The PIAMA cohort

The PIAMA birth cohort is a Dutch population-based study that started in 1996/97 with 3963 new-borns and has been extensively described elsewhere [8]. Follow-ups were conducted at the child's age of 3 months, yearly until age 8, and then at ages 11, 14, 16 and 17 years. Medical examinations with measurements of lung function including FEV_1_ and FVC and anthropometric characteristics such as weight and height were conducted at ages 8, 12 and 16. At age 16, lung function measurements were obtained in 721 participants. Both the Masterscreen pneumotachograph (CareFusion, Yorba Linda, CA, USA) and Easy One spirometers (NDD Medical Technologies, Inc., Switzerland) were used to measure FEV_1_ and FVC at age 16 in two centres, Groningen and Utrecht respectively. We applied the correction equation in the current study to lung function data from the PIAMA cohort measured at age 16.

Ethical approval of the current study was obtained from medical ethical review board from University Medical Center Groningen (ref no. M17.220613) and all volunteers provided consent to participate.

### Spirometers

We used two EasyOne spirometers (NDD Medical Technologies, Inc., Switzerland) and the Jaeger Masterscreen pneumotachograph spirometer (CareFusion, Yorba Linda, CA, USA).

The Masterscreen pneumotachograph is one of the most widely used pulmonary function systems. It measures lung volumes indirectly with a pneumotachograph using the pressure difference over a small, fixed resistance, offered by a fine metal mesh [9]. In brief, it measures the pressure drop when a patient blows into the device. The pressure drop divided by the resistance of the pneumotachograph yields the flow, which can be transformed into a volume by time integration [10]. It is sensitive to temperature, humidity and atmospheric pressure of surrounding air and therefore requires constant calibration.

The EasyOne spirometer is a handheld standalone flow-sensing instrument that requires no calibration though calibration can be checked with a syringe [11]. Unlike the Masterscreen pneumotachograph, the EasyOne spirometer incorporates an ultrasonic flow sensor to measure the flow of air in and out of the patients’ lungs. Ultrasonic flow measurements are independent of gas composition, pressure, temperature, and humidity and therefore inaccuracy is reduced due to the mentioned factors [12].

### Statistical analysis

Sample size calculations were performed based on a standard deviation (SD) for FEV_1_ of 0.5 L. With a significance level of 0.05, 44 volunteers were required to detect a mean difference of 0.3 L between the spirometers with 80% power.

Correlations and agreement between spirometry measurements performed with the different spirometers were assessed with scatterplots, Pearson correlation coefficients and Bland and Altman plots [13]. Significance of differences between spirometers (within persons) was tested with paired t-tests.

In the absence of a gold standard, we computed the percent predicted FEV_1_ and FVC according to sex, age, height, and ethnicity based on reference regression equations developed by the Global Lung Function Initiative (GLI) [14] to assess which of the two spirometers most likely gives a better estimate of the lung function.

Moreover, we used the data from the first test series to provide a correction equation by regressing measurements from the Masterscreen pneumotachograph on the measurements obtained by the EasyOne1 spirometer as follows:

$$ {FEV}_1 Masterscreen=\alpha +{\beta}^{\ast }{FEV}_1 EasyOne1 $$

$$ FVCMasterscreen=\alpha +{\beta}^{\ast } FVCEasyOne1 $$

The regression coefficients can be used to correct for systematic differences in epidemiological analyses and we showed this by applying the equation to lung function data from the PIAMA birth cohort collected at age 16. Data were analysed using SAS version 9.4 (The SAS Institute, Cary, NC, USA).

## Results

Table 1 shows characteristics of the volunteers that participated in the two series of spirometer comparisons. On average, the FEV_1_ and FVC as measured by the Masterscreen pneumotachograph were significantly higher than the FEV_1_ and FVC as measured by the EasyOne1 spirometer (FEV_1_: 3.78 L vs 3.54 L, mean difference 0.24 L, *p*-value < 0.0001; FVC: 4.78 L vs 4.41 L, mean difference 0.37 L, *p*-value < 0.0001). The 0.24 L and 0.37 L mean differences, correspond to a 6.3% decrease in FEV_1_ switching from the Masterscreen pneumotachograph to the EasyOne1 spirometer and 8.4% decrease in FVC switching from the Masterscreen pneumotachograph to the EasyOne1 spirometer respectively. Differences in FEV_1_ and FVC between the two EasyOne spirometers were small i.e. FEV_1_: 3.50 L vs 3.46 L with a mean difference of 0.03 L, p-value < 0.003 and FVC: 4.31 L vs 4.27 L mean difference, 0.04 L, *p*-value < 0.003, respectively. The mean differences correspond to a 1.1% decrease in FEV_1_ switching from the EasyOne1 to the EasyOne2 spirometer and 0.9% decrease in FVC switching from the EasyOne1 to the EasyOne2 spirometer (Tables 1 and 2). The observed differences between the spirometers were similar in males and females and in younger and older volunteers (Table 2).

**Table 1:** Study population characteristics

Masterscreen vs EasyOne1	Overall (*N* = 49)	Males (*N* = 15)	Females(*N* = 34)
Age (years) – mean (SD)	30.2 (10.9)	29.2 (10.8)	30.8 (11.1)
Age ≤ 30 years – N (%)	32 (65)	12 (66)	29 (67)
Ethnicity-N (%)			
Caucasian	49 (100)	15 (100)	34 (100)
Weight (Kg) – mean (SD)	68.9 (11.3)	72.6 (11.9)	66.9 (10.6)
Height (m) – mean (SD)	1.74 (8.32)	1.81 (6.46)	1.70 (6.57)
FEV_1_Masterscreen (L) – mean (SD)	3.78 (0.63)	4.38 (0.62)	3.51(0.43)
FEV_1_EasyOne 1 – mean (SD)	3.54 (0.60)	4.11 (0.57)	3.29 (0.42)
FVC Masterscreen (L) – mean (SD)	4.78 (0.85)	5.77 (0.76)	4.35 (0.42)
FVCEasyOne1 (L) – mean (SD)	4.41 (0.83)	5.35 (0.74)	4.01 (0.44)
FEV_1_Masterscreen mean (SD) percent predicted	98.3 (11.1)	93.6 (10.5)	100.4 (10.8)
FEV_1_EasyOne1 mean (SD) percent predicted	92.3 (10.8)	87.9 (9.9)	94.2 (10.7)
FVC Masterscreen mean (SD) percent predicted	103.7 (10.5)	101.2 (11.4)	104 (10.1)
FVC EasyOne1 mean (SD) percent predicted	95.5 (10.5)	93.8 (12.2)	96.2 (9.87)
EasyOne1 vs EasyOne2	Overall (*N* = 49)	Males (*N* = 17)	Females (*N* = 32)
Age (years) - mean (SD)	35.1 (11.4)	32.8 (10.4)	37.4 (12.1)
Age ≤ 30 years – N (%)	23 (46)	11 (47)	12 (52)
Ethnicity- N (%)			
Caucasian	43 (88)	15 (88)	28 (88)
Asian	4 (8)	2 (12)	2 (6)
Other	2 (4)	0 (0)	2 (6)
Weight (Kg) – mean (SD)	68.1 (10.1)	76.1 (9.3)	63.7 (7.7)
Height (m) – mean (SD)	1.71 (0.11)	1.82 (0.72)	1.65 (0.86)
FEV_1_EasyOne 1(L) – mean (SD)	3.50 (0.85)	4.33 (0.63)	3.05 (0.58)
FEV_1_EasyOne 2 (L) – mean (SD)	3.46 (0.84)	4.27 (0.62)	3.03 (0.58)
FVCEasyOne1(L) – mean (SD)	4.31 (1.05)	5.45 (0.64)	3.71 (0.65)
FVCEasyOne2 (L) – mean (SD)	4.27 (1.04)	5.38 (0.65)	3.68 (0.66)
FEV_1_ EasyOne1 mean (SD) percent predicted	95.8 (11.1)	92.8 (12.2)	97.4 (10.2)
FEV_1_ EasyOne2 mean (SD) percent predicted	94.8 (11.2)	91.5 (12.1)	96.5 (10.5)
FVCEasyOne1mean (SD) percent predicted	97.4 (9.9)	96.1 (11.1)	98.1 (9.3)
FVCEasyOne2mean (SD) percent predicted	96.5 (10.2)	94.8 (11.1)	97.4 (9.7)

**Table 2:** Mean differences (with confidence intervals): Masterscreen vs EasyOne1 and EasyOne1 vs EasyOne2, overall and by age and sex

		FEV_1_ (L)		FVC (L)	
	N	Mean diff.	95% CI	Mean diff.	95% CI
Masterscreen –EasyOne1					
Overall	49	0.24	(0.19;0.26)	0.37	(0.33; 0.41)
≤ 30 years	32	0.23	(0.18; 0.27)	0.37	(0.31; 0.42)
> 30 years	17	0.23	(0.17; 0.29)	0.38	(0.33; 0.44)
Males	15	0.26	(0.18; 0.35)	0.42	(0.31; 0.53)
Females	34	0.21	(0.18; 0.24)	0.35	(0.31; 0.39)
EasyOne1 –EasyOne2					
Overall	49	0.03	(0.01;0.06)	0.04	(0.01; 0.06)
≤ 30 years	23	0.03	(−0.00; 0.08)	0.04	(0.00; 0.08)
> 30 years	26	0.03	(0.00; 0.06)	0.03	(−0.00; 0.07)
Males	17	0.06	(0.00; 0.11)	0.06	(0.01; 0.12)
Females	32	0.02	(0.00; 0.05)	0.02	(− 0.00;0.05)

**Fig. 1:**
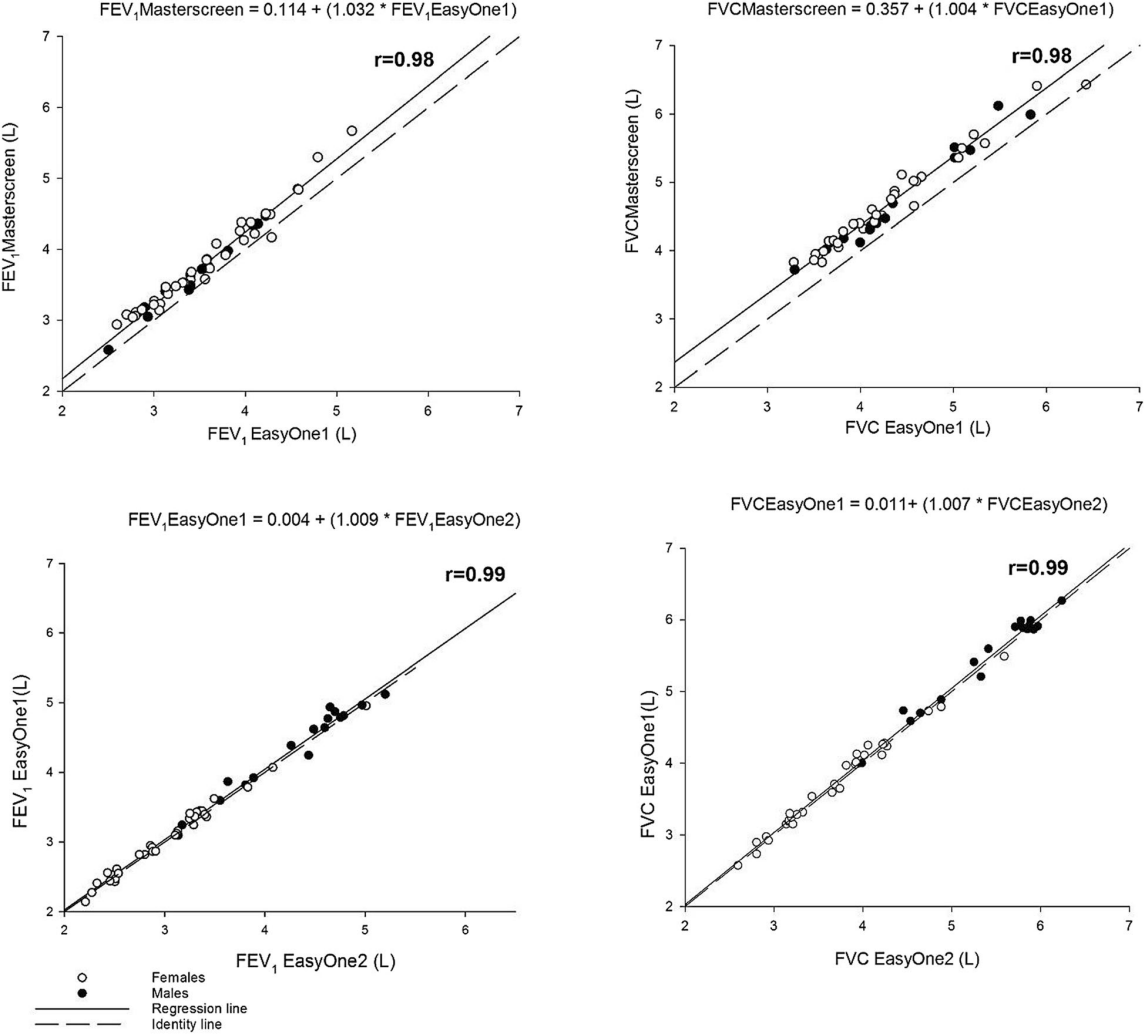
Correlation between measurements from the first comparison series (Masterscreen and EasyOne1 spirometer, upper panels) and the second series (EasyOne1 spirometer from the first series and another EasyOne2 spirometer of the same generation, lower panel)

**Fig. 2:**
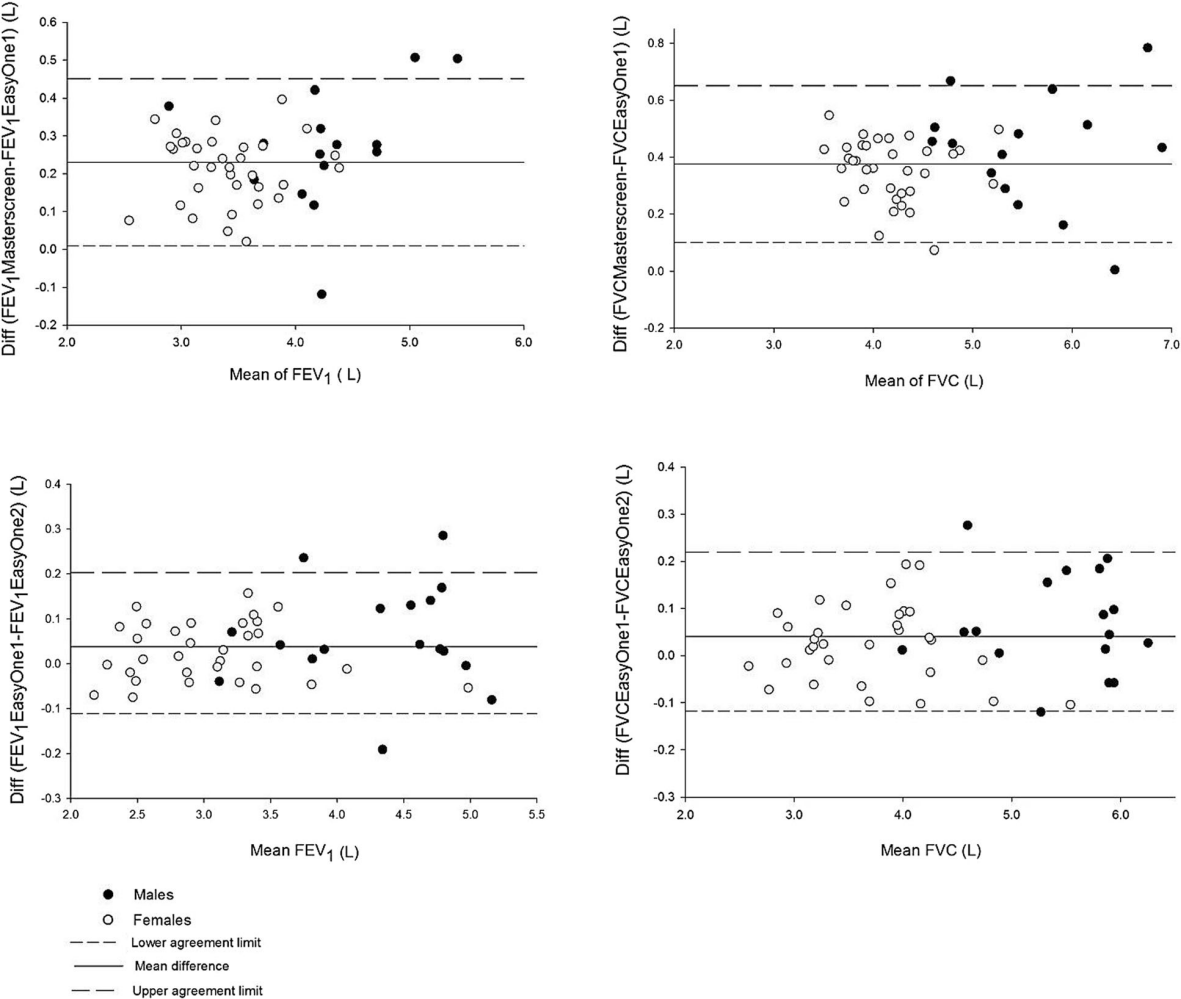
Bland and Altman plots of FEV_1_ and FVC measurements from the first test series (Masterscreen and EasyOne1 spirometer, upper panels) and the second test series (EasyOne1 spirometer from the first series and another EasyOne2 spirometer of the same generation, lower panel)

Measurements were highly correlated (r = 0.98 for the first test series and r = 0.99 for the second test series for both FEV_1_ and FVC) indicating a strong linear relationship, which deviates from identity (Fig. 1) for FEV_1_ (but not FVC) in the first test series, but not for the second test series. The Bland and Altman plots show that the mean differences are consistently larger than zero indicating a systematic difference between the two spirometers with the Masterscreen pneumotachograph consistently producing higher values than the EasyOne1. There was no systematic difference between the two EasyOne1 and EasyOne2 measurements (Fig. 2).

Using the GLI reference equations, the percent predicted for the Masterscreen pneumotachograph was close to 100% (98.3% for FEV_1_ and 103.7% for FVC), but less so for the EasyOne1 (92.3% for FEV_1_ and 95.5% for FVC).

Regression of the measurements from the Masterscreen pneumotachograph on the EasyOne1 measurements produced the following regression equations (Fig. 1):

$$ {FEV}_1 Masterscreen=0.114\ (0.05)+1.032\ {(0.01)}^{\ast }\ {FEV}_1 EasyOne1 $$

$$ FVCMasterscreen=0.357\ (0.05)+1.005{(0.01)}^{\ast }\  FVCEasyOne1 $$

The above regression equations indicate that an increase of 1 L in the EasyOne1 measurements is associated with an estimated average increase of 1.032 L for the FEV_1_ and 1.005 L for the FVC in the Masterscreen pneumotachograph measurements.

Table 3 shows the mean of FEV_1_ and FVC as measured in the PIAMA birth cohort at the age of 16 years, before and after correction for the systematic differences. The mean difference reduces from 0.37 L to 0.13 L for FEV_1_ and 0.44 L to 0.07 L for FVC after correction.

**Table 3:** Means of corrected lung function measurements from PIAMA lung function data

	Uncorrected Mean (95% CI)	Corrected Mean (95% CI)
Overall FEV1 (L)	3.81 (3.75; 3.86)	3.94 (3.89; 4.00)
FEV1_EasyOne1 (L)	3.65 (3.58; 3.72)	3.88 (3.81; 3.95)
FEV1_Masterscreen (L)	4.03 (3.95; 4.11)	4.03 (3.95; 4.11)
Mean difference (L)	−0.37 (− 0.47; − 0.26)	−0.13 (− 0.24; − 0.03)
Overall FVC (L)	4.48 (4.42; 4.55)	4.70 (4.64; 4.77)
FVC_EasyOne1 (L)	4.30 (4.21; 4.38)	4.67 (4.59;4.76)
FVC_Masterscreen (L)	4.74 (4.64; 4.84)	4.74 (4.64; 4.84)
Mean difference (L)	−0.44 (− 0.56; − 0.31)	−0.07 (− 0.19; 0.07)

## Discussion

We compared FEV_1_ and FVC measurements from two different, widely used spirometers, the EasyOne and Masterscreen pneumotachograph and found that the EasyOne spirometer provided on average systematically lower measurements than the Masterscreen. We also investigated the agreement between two EasyOne spirometers of the same generation and found that measurements were comparable, but with a small significant difference.

In epidemiological studies, lung function measurements can be performed using more than one spirometer of the same type or different types. This study showed a systematic difference between two types of spirometers used in the PIAMA birth cohort study [15]. We conducted this experiment in healthy volunteers for which the mean percent predicted FEV_1_ and FVC was expected to be close to 100%. Based on reference equations provided by the GLI [14], for none of the spirometers the mean percent FEV_1_ and FVC was exactly 100%, but percentages were closer to 100% for the Masterscreen pneumotachograph than the EasyOne1 especially for FEV_1_. The lower percent predicted lung function for the EasyOne1 suggests that the EasyOne spirometer may be more likely to overestimate the percentage of subjects with a clinically low lung function in a setting where different spirometers are used. This has been previously demonstrated in a comparison involving the EasyOne spirometer and a water-sealed spirometer (Collins, Stead-Wells) where underestimated values of both FEV_1_ and FVC from the EasyOne spirometer and consequently higher prevalence rates of airway obstruction were observed [16]. It is important to note that the GLI reference equations are not universally applicable. However, these equations are based on an extensive database and studies in the Netherlands have shown that measurements in the Dutch population generally agree with the GLI references values in adults [17]. We therefore believe these equations are most likely suitable for our current study population as the Masterscreen-EasyOne comparison population was 100% Dutch. It is advised that regardless of which reference equations are used, clinical decisions should never be based solely on lung function test results but backed up with complementary laboratory clinical and physical findings [18].

Several studies have conducted similar experiments comparing different types of spirometers, handheld/office and standard laboratory spirometers both in clinical and research settings [2–4, 19–22], with the comparisons also used as quality control procedure in international multicentre epidemiological studies [23, 24]. High correlations were observed throughout these studies, but significant systematic differences between spirometers in some of the studies [2, 19, 20] suggest that measurements from different spirometers are not always comparable. Kunzli et al. [4] conducted a study comparing eight flow sensing spirometers of the same type (Sensormedics 2200) and found that the new generation of Sensormedics (V_max_) gave systematically lower results than the older generation. Based on this comparison, an informed decision on choice of spirometers to use for their follow up study was made by excluding the new generation spirometers in the SALPADIA cohort. Similar practical changes were made in another study based on a similar comparison [23]. Small systematically lower FVC and FEV_1_ at follow-up, may eventually translate into erroneous deficits of lung function in the studied population, leading to erroneous conclusions about the effect of environmental, biologic or life-style factors on lung function changes [2]. Use of different types of lung function spirometers in the same study can be less detrimental if comparability is established and if necessary any systematic differences corrected.

The source of the observed differences between the Masterscreen pneumotachograph and the EasyOne spirometer is unclear. The Masterscreen pneumotachograph was routinely calibrated for each session as per requirement. The EasyOne spirometers are made to require no calibration but were occasionally checked using a calibration syringe. Both spirometers were therefore thoroughly checked as regards calibration such that chances that the observed differences are due to calibration differences are minimal. However, the following limitations should be considered: two experienced technicians performed the first test series (one with the Masterscreen pneumotachograph and one with the EasyOne) and one of them performed all measurements of the second test series. We designed the comparison of the Masterscreen pneumotachograph and EasyOne spirometers such that different technicians operated the different spirometers to imitate a real multicentre study. While the technicians were highly trained and experienced, due to the study design it was impossible to disentangle differences between spirometers from differences between technicians. Consequently, part of the observed difference between spirometers may be attributable to differences between technicians. The provided correction equation thus simultaneously corrects for the technician and device effect and may not be generalizable to other studies where different technicians are involved. However, it is expected that the calibration method can be applied accordingly. We were not able to assess the external validity of the correction for spirometry measurements outside the PIAMA population, but it has been used before to correct spirometry measurements [6] and the method has been validated in other fields of epidemiology [25]. We used self-reported instead of measured height and weight for the in total 98 volunteers that participated in the comparisons of the spirometers. Since spirometers were compared within persons, and consequently height and weight did not differ between the spirometers that were compared within a series, this does not affect the observed differences between spirometers. Self-reported height might be a source of bias when applying the GLI equations as height values may be over−/underreported. Weight is not used in the GLI equations to estimate percent predicted lung function and therefore poses no risk of bias. Studies of the agreement between self-reported and measured weight and height provided inconsistent results, some suggested good agreement [26, 27], while others reported significant discrepancies mainly in overweight/obese individuals [28, 29]. It is also not clear to what extent the systematic differences between the two spirometers can be attributed to hardware as computer software has been identified as another as major source of discrepancies between spirometers [30].

The strength of this study is that the order of the spirometers was randomized to minimize influences of personal characteristics and differences due to study design. We observed high precision of the regression parameter estimates, which highly suggests that the sample size in our experiment is not a concern.

## Conclusion

We observed systematic differences between lung function measurements from two spirometers of different types. Epidemiological researchers need to be aware of these potential systematic differences and correct for them in the analyses using methods such as regression calibration.

## Abbreviations

FEV_1_: Forced expiratory flow in 1 second
FVC: Forced vital capacity
GLI: Global Lung Function Initiative
PIAMA: Prevention and incidence of asthma and mite allergy
